# Non-alcoholic fatty liver disease combined with rheumatoid arthritis exacerbates liver fibrosis by stimulating co-localization of PTRF and TLR4 in rats

**DOI:** 10.3389/fphar.2023.1149665

**Published:** 2023-06-06

**Authors:** Shengpeng Zhang, Peng Zhu, Jianan Yuan, Kunming Cheng, Qixiang Xu, Wei Chen, Zui Pan, Yongqiu Zheng

**Affiliations:** ^1^ School of Pharmacy, Wannan Medical College, Wuhu, China; ^2^ Boster Biological Technology Co., Ltd., Wuhan, China; ^3^ College of Nursing and Health Innovation, The University of Texas at Arlington, Arlington, TX, United States

**Keywords:** non-alcoholic fatty liver disease, rheumatoid arthritis, polymerase I and transcript release factor, TLR4, PTRF, PI3K/AKT signaling

## Abstract

Rheumatoid arthritis (RA) has a high prevalence in patients with non-alcoholic fatty liver disease (NAFLD); however, the underlying mechanism is unclear. To address this, our study established a rat model with both NAFLD and RA by feeding a high-fat diet (HFD) and administering intradermal injection of Freund’s complete adjuvant (FCA) with bovine type II collagen. Collagen-induced RA (CIA) was confirmed by hind paw swelling and histological examination. The histomorphological characteristics of NAFLD were evaluated by Masson’s trichrome and hematoxylin-eosin staining. The development of NAFLD was further evaluated by measuring serum concentrations of triglyceride (TG), total cholesterol (T-CHO), alanine aminotransferase (ALT), aspartate aminotransferase (AST), and lipopolysaccharide (LPS). The results showed that HFD feeding exacerbated secondary inflammation in CIA rats, whereas FCA/bovine type II collagen injection increased serum levels of ALT, AST, TG, T-CHO, and LPS and exacerbated hepatic fibrosis in both normal and NAFLD rats. Interestingly, NAFLD + CIA significantly promoted the expression of PTRF, a caveolae structure protein involved in hepatic lipid metabolism and affecting downstream signaling of Toll-like receptor 4 (TLR4) and PI3K/Akt activation. High resolution confocal microscopy revealed increased PTRF and TLR4 co-localization in hepatic small vessels of NAFLD + CIA rats. AAV9-mediated PTRF knockdown inhibited TLR4 signaling and alleviated hepatic fibrosis in NAFLD + CIA rats. Together, these findings indicate that NAFLD combined with CIA causes synovial injury and enhances non-alcoholic fatty liver fibrosis in rats. PTRF could attenuate the symptoms of NAFLD + CIA likely by affecting TLR4/PTRF co-expression and downstream signaling.

## 1 Introduction

Rheumatoid arthritis (RA) is a common persistent joint inflammation characterized by joint damage, deformities, and dysfunction, affecting 0.5%–1% of the worldwide population (Guo et al., 2020). As a systemic inflammatory disease, it often affects extra-articular organs. Although liver injury is not considered a major pathological feature of RA, abnormal liver performance, including elevated alkaline phosphatase, fluctuating with disease activity, has been observed in 18%–50% of patients with RA (Selmi et al., 2011). A retrospective study of 188 RA cases reported that 65% of the unselected patients with RA had abnormal liver biopsies, 50% had chronic portal inflammatory infiltrate entering the portal tract and moderate necrosis, and 25% had fatty liver changes (Ruderman et al., 1997). Another study of 846 patients with RA indicated that 42 patients who were treated with methotrexate had persistent elevation of transaminases (Mori et al., 2018). Further ultrasound and histological analyzes revealed non-alcoholic steatohepatitis (NASH) as the most prevalent pattern of liver injury in these patients with RA.

Non-alcoholic fatty liver disease (NAFLD) is a common chronic liver disease worldwide, especially in the United States, with a prevalence of 20%–30% (Bellentani et al., 2010). Dyslipidemia, body fat accumulation, and oxidative stress are closely associated with NAFLD (Maurice and Manousou, 2018). The accumulation of lipids in the liver can cause excessive inflammation, oxidation, and fibrosis, and eventually progress to non-alcoholic cirrhosis and liver cancer (Farrell and Larter, 2006). The insulin signaling pathways, especially the phosphatidylinositol-3-kinase (PI3K)/protein kinase B (Akt) pathway, appear to be dysregulated during the development of NAFLD. While clinical evidence has shown a strong prevalence of NAFLD in patients with RA, the underlying pathological mechanism remains largely unknown, and effective treatment for these patients is lacking (Sanyal et al., 2015). Hence, there is an urgent need to identify therapeutic targets for patients with both NAFLD and RA.

Toll-like receptor 4 (TLR4) plays an important role in immune system activation and is an established receptor for lipopolysaccharides (LPSs). Intriguingly, hepatocytes express TLR4 and the machinery for the TLR4 signaling pathway. Liver-specific TLR4 knockout mice appeared to be resistant to diet-induced NAFLD, suggesting that the TLR4 signaling pathway in hepatocytes plays a fundamental role in the development of NAFLD (Shen et al., 2018). These mice also exhibited enhanced insulin sensitivity and ameliorated hepatic steatosis after a high-fat diet (HFD) challenge (Jia et al., 2014). Additionally, several other reports have indicated that the TLR4 pathway is critically involved in NASH progression in different animal models (Zhai et al., 2004; Csak et al., 2011). The hepatic PI3K/Akt axis appears to be the downstream primary insulin signaling pathway affecting NAFLD development (Matsuda et al., 2013).

Our group previously showed that the TLR4 signaling pathway is stimulated by polymerase 1 and transcript release factor (PTRF), also known as cavin-1, a conserved structural protein involved in the formation of caveolae (Zhou et al., 2021). TLR4 physically binds to PTRF in caveolae of the plasma membrane at the basal level, and PTRF is required for TLR4 signaling assembly, which is especially stimulated by LPS (Zheng et al., 2013). It is well established that PTRF plays important roles in regulating cell functions, including proliferation, mitochondrial function, migration, and senescence (Calvo et al., 2001; Fernandez-Rojo and Ramm, 2016; Bai et al., 2020). In hepatocytes, PTRF can control hepatic lipid metabolism and participate in some key pathways during the progression of hepatic diseases (Fernandez-Rojo et al., 2013; Ding et al., 2014). However, the exact role of TLR4 signaling in patients with RA with NAFLD and whether it is regulated by PTRF remain to be elucidated. In this study, a compound rat model was established by HFD feeding to induce NAFLD and by intradermal injection of Freund’s complete adjuvant (FCA) and bovine type II collagen to induce RA. The role of the PTRF-TLR4 signaling axis was then studied in this compound model.

## 2 Materials and methods

### 2.1 Animals

Male Sprague–Dawley rats (5–6 weeks old, 145–165 g) were purchased from Hunan Silaike Jingda Technology Co., Ltd. (No: SCXK; Hunan; 2019-0004) and acclimatized for 7 d. The rats (5 rats per cage) were kept at a controlled room temperature (23°C ± 1°C) and relative humidity (55% ± 5%) in the SPF Lab of Wannan Medical College, and water was available *ad libitum*. All rats were handled for experimental protocols in accordance with the National Institutes of Health regulations program, and the study was approved by the Institutional Animal Care and Use Committee of Wannan Medical College (Approval number: LLSC-2022-216).

### 2.2 Model establishment

**FIGURE 1:**
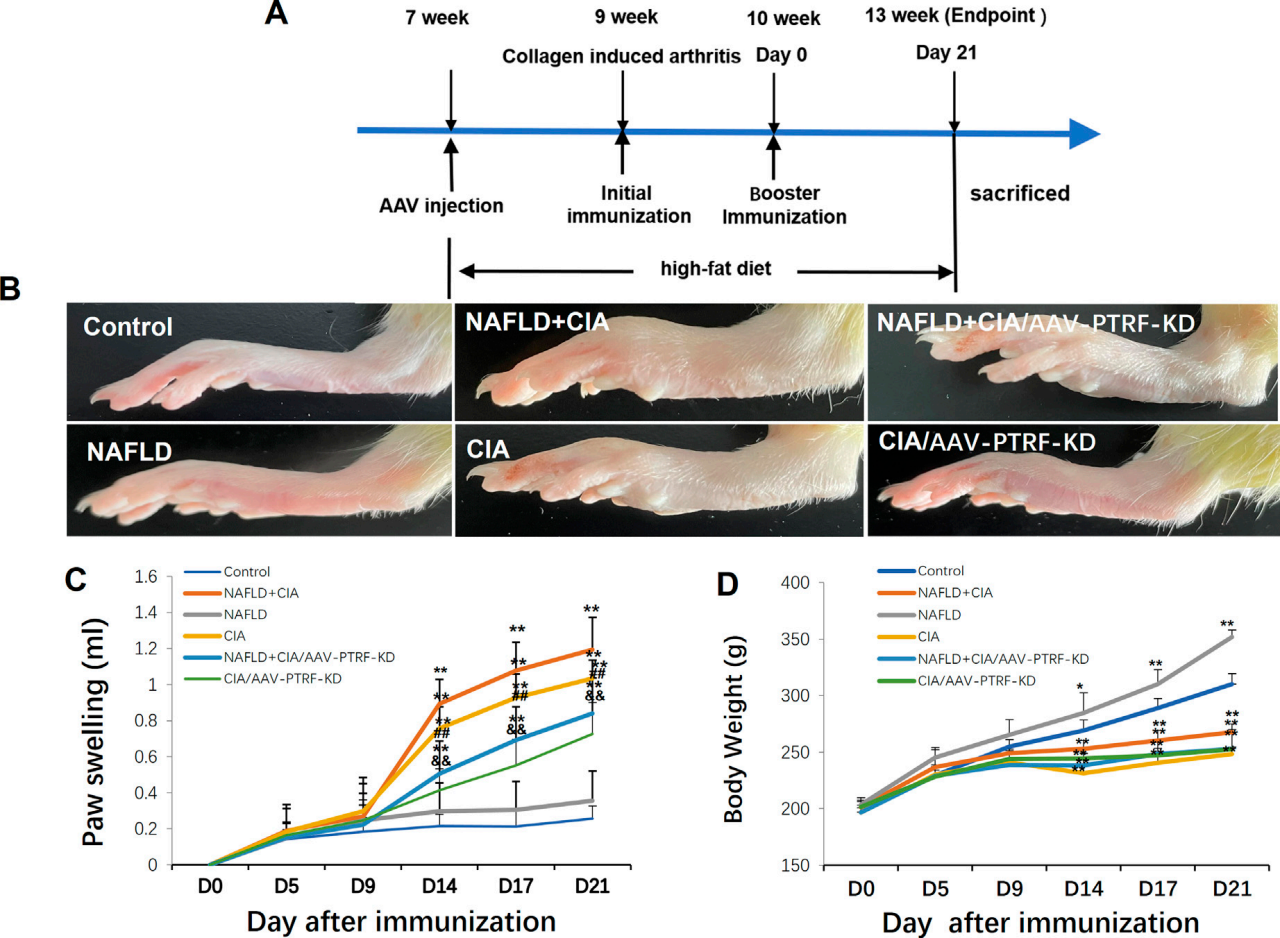
Effect of HFD and/or FCA/bovine type II collagen on synoviocyte proliferation in CIA and/or NAFLD rats. **(A)** Experimental protocol. **(B)** Images of the swelling of the secondary paw of CIA and NAFLD + CIA rats. **(C)** Degree of swelling of the non-injected hind paw (in mL) was measured. **(D)** Body weight changes in CIA rats with or without NAFLD. Values are presented as mean ± SD, n = 10. **p < 0.01 *vs*. Control group, ^##^ *p* < 0.01 *vs*. NAFLD + CIA group, ^&&^P < 0.01 *vs*. CIA group.

The compound animal model of NAFLD (Zhu et al., 2023) + collagen-induced RA (CIA) (Wang et al., 2018; Guo et al., 2020) was established using the following process. Forty unselected Sprague-Dawley rats were grouped as follows: normal (control) group, CIA group, NAFLD model group, and NAFLD + CIA group (10 rats per group). The NAFLD model group and NAFLD + CIA group rats received an HFD (40 kcal% fat, 20 kcal% sucrose, and 2% cholesterol) for 6 weeks to develop NAFLD. The HFD was provided by Xietong Bio-engineering Co., Ltd. Thereafter, 1 g/L of a mixture of CFA and bovine type II collagen (Sigma–Aldrich, MO, United States; ratio of 1:1) in a 0.1-ml emulsifier was subcutaneously injected into the base of the tail in the CIA and NAFLD + CIA groups. The rats were further immunized with 0.1 ml of emulsifier after 7 d via subcutaneous injection into the proximal one-third of the tail. Clinical severity was evaluated by measuring the footpad thickness using a dial-gauge caliper. Paw swelling (ml) was calculated by subtracting the paw volume (left hind paw) at d0 (Figure 1A) (Ahmad Khan et al., 2020).

### 2.3 Adeno-associated virus (AAV) serotype 9 (AAV9)-mediated gene knockdown *in vivo*

For additional knockdown studies, the shPTRF sense oligonucleotide sequence 5′-GCC​AGA​TAA​AGA​AAC​TGG​AGG​TCA​A-3ʹ and the shPTRF antisense oligonucleotide sequence 5′-TTG​ACC​TGG​AGT​TTC​TTT​ATC​TGG​C-3ʹ were annealed and cloned into the GV594-U6-MCS-CAG-firefly_Luciferase vector. An unrelated sequence was used as the control. PTRF knockdown and control recombinant AAV9-luciferase vectors were constructed (GENECHEM Biotech). Another 25 rats were randomly divided into five groups (five rats per group): control, NAFLD + CIA/AAV9-NC, NAFLD + CIA/AAV9-PTRF-KD, NAFLD/AAV9-NC, and NAFLD/AAV9-PTRF-KD. The administration procedures were performed as described previously (5 × 10^10^ physical particles/PBS per rat; intravenous injection via the tail vein of the rats in the NAFLD + CIA/AAV9-NC, NAFLD + CIA/AAV9-PTRF-KD, NAFLD/AAV9-NC, and NAFLD/AAV9-PTRF-KD groups) (Fang et al., 2019).

### 2.4 Histopathological analysis

For histopathological analysis, identical liver tissue segments were collected from each rat. Tissue segments were kept in 4% paraformaldehyde, embedded in paraffin, and sectioned for routine hematoxylin-eosin (HE) and Masson’s trichrome staining. The HE- and Masson’s trichrome-stained sections were examined under a light microscope and photographed using an Olympus camera system (BX51) to analyze the degree of hepatic steatosis and inflammation. Confocal images were taken with a Zeiss LSM-510 microscope. ImageJ software was used to calculate the collagen volume fraction of Masson’s trichrome staining. The Masson-positive area/total area (%) values were depicted as bar graphs.

### 2.5 Biochemical analysis

The levels of biochemical markers [alanine transaminase (ALT), aspartate transaminase (AST), triglyceride (TG), total cholesterol (T-CHO), and LPS] in the serum and the content of hydroxyproline (Hyp) in 10% liver homogenate were determined using the respective detection kits (NanJing JianCheng Biological Engineering).

### 2.6 Immunofluorescence

Liver issue slices embedded in paraffin (5-μm thick) were prepared for immunofluorescence analysis. The TLR4 antibody (1:50, Thermo Invitrogen, Shanghai, China), CD31 antibody (1:50, Abcam, Shanghai, China), PTRF antibody (1:50, Thermo Invitrogen, Shanghai, China), and α-SMA antibody (1:50, Abcam, Shanghai, China) were used as primary antibodies, and the FITC-conjugated goat anti-mouse antibody (1:500, Abcam, Shanghai, China) and CY3-conjugated goat anti-rabbit antibody (1:500, Abcam, Shanghai, China) were used as the secondary antibodies; 4′,6-diamidino-2-phenylindole (DAPI) was used as a nuclear indicator. The slides were subsequently visualized using a confocal laser-scanning microscope (LSM 880, Carl Zeiss, Oberkochen, Germany).

### 2.7 Western blot analysis

Total proteins of hepatic tissues were extracted using a lysis buffer containing proteases, RIPA, and phosphatase inhibitors. Proteins were separated by electrophoresis and further electro-transferred onto a polyvinylidene fluoride membrane. Non-specific binding was blocked by adding 5% bovine serum albumin for 2 h at 24°C ± 2°C. Next, the membrane was incubated overnight at 4°C with anti-Akt (1:1000, Cell Signaling), anti-p-Akt (1:1000, Thermo Invitrogen, Shanghai, China), anti-PTRF (1:1000, Thermo Invitrogen, Shanghai, China), anti-PI3K) (1:1000, Abcam, Shanghai, China), and anti-GAPDH (1:1000, Cell Signaling, Boston, United States) primary antibodies in PBS + Tween buffer. The blots were then detected using an enhanced chemiluminescence kit (GE Biosciences, City of Saint Louis), United States), and the Bio-Rad Image LabTMVersion 6.0 software was used to analyze the density of the blots.

### 2.8 Statistical analysis

The data are presented as mean ± standard deviation (SD) and were analyzed using the SPSS software 23.0. The data were analyzed using a one-way analysis of variance, followed by *post hoc* Dunnett’s t-test for multiple comparisons. A nonparametric test was used to compare band density values between the groups. Differences were considered statistically significant at *p* ≤ 0.05.

## 3 Results

### 3.1 HFD enhances secondary arthritis injury induced by FCA combined with bovine type II collagen in CIA rats

**FIGURE 2:**
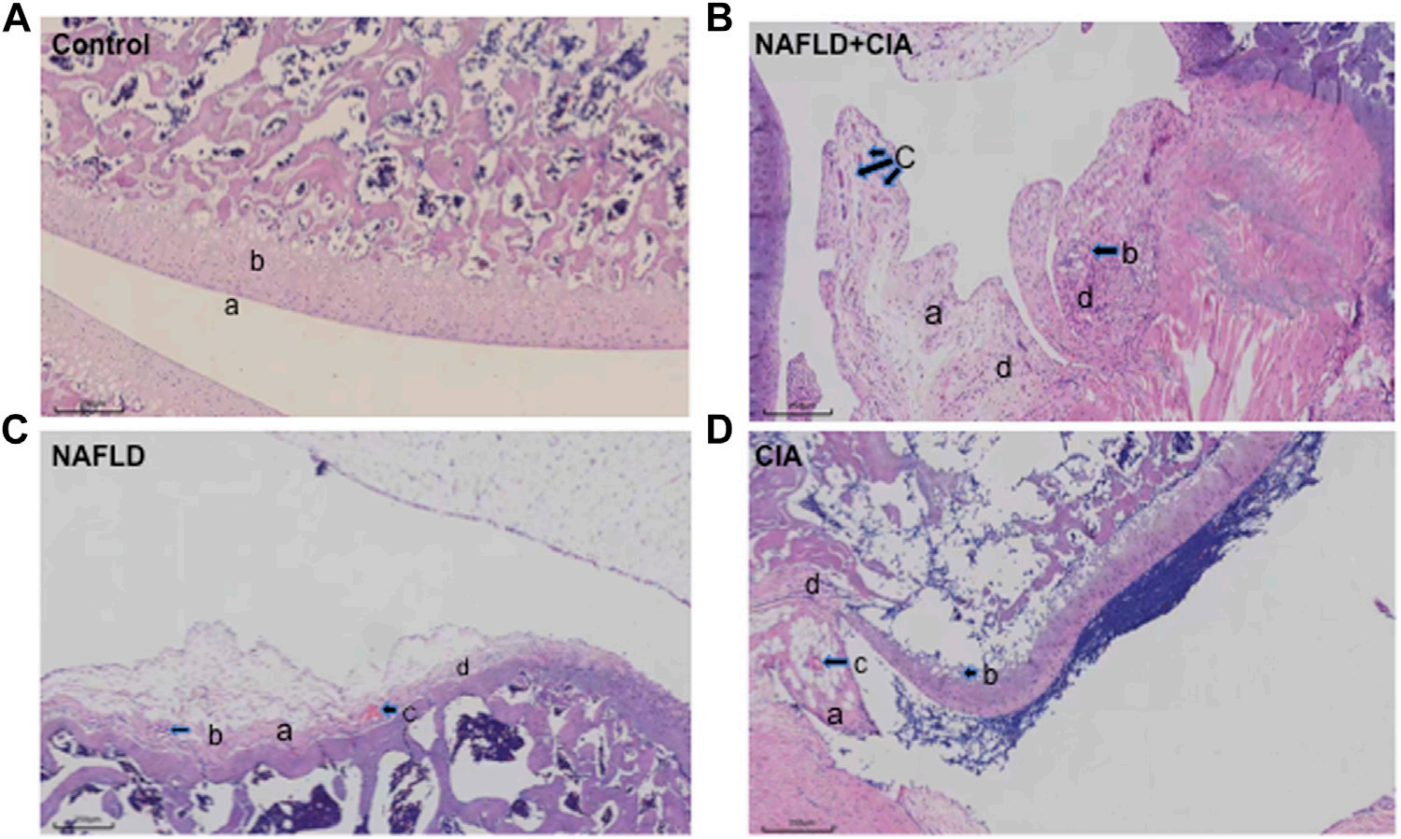
Histopathologic changes of the synovium in CIA and NAFLD rats. **(A)** In normal rats, synoviocytes were in monolayers (a) and articular cartilage was normal (b). **(B)** In CIA + NAFLD rats, serious proliferation of synoviocytes (a) and destruction of articular cartilage (b), with new blood vessels (or pannus) (c) and infiltration of inflammatory cells (d) were observed. **(C)** In NAFLD rats, there was slight destruction of articular cartilage (a), new blood vessels (b), pannus (c), and infiltration of inflammatory cells (d). **(D)** In CIA rats, destruction of articular cartilage (a), new blood vessels (b), pannus (c), and infiltration of inflammatory cells (d) were observed (b). Scale bar = 250 µm.

Hind paw secondary swelling scores showed that secondary arthritis became significant at Day 14 and lasted until Day 21, after immunization in both the CIA and NAFLD + CIA groups, but was absent in the control or NAFLD group (Figures 1B, C). In NAFLD + CIA rats, the swelling was slightly increased on Days 14–21 compared with that in CIA rats, but the difference did not reach statistical significance (Figure 1C). As expected, HFD increased body weight in NAFLD rats compared with that of the control group (*p* < 0.01, Figure 1D). Interestingly, the body weight of CIA rats decreased compared with that of the control group, even the HFD failed to maintain the body weight, as evidenced by the fact that the body weight of CIA + NAFLD rats decreased significantly on Days 14, 17, and 21, compared with that of the control rats (*p* < 0.01, Figure 1D). To further explore the pathophysiological mechanisms of NAFLD on CIA, AAV-9 containing a specific shRNA targeting PTFR for gene knockdown was used. The rats were transfected with the AAV-PTRF knockdown (AAV-PTRF-KD) vector through intravenous tail injection. Paw swelling induced by FCA was alleviated by AAV-PTRF-KD in CIA rats and CIA + NAFLD rats. However, AAV-PTRF-KD had no effects on body weight in both CIA rats and CIA + NAFLD rats (Figures 1C, D). Furthermore, NAFLD prominently enhanced synovial inflammatory lesions in CIA rats but only induced slight synovial inflammatory lesions in control rats (*p* < 0.01, Figure 2). The proliferation of the synovium and destruction of articular cartilage with new blood vessels (or pannus) were observed in CIA rats. In CIA + NAFLD rats, synovial hyperplasia and pannus were promoted, and the destruction of articular cartilage was more severe. HFD also induced slight destruction of the pannus and articular cartilage (Figure 2).

### 3.2 Serum levels of ALT, AST, TG, T-CHO, and LPS were increased in rats with NAFLD + CIA

**FIGURE 3:**
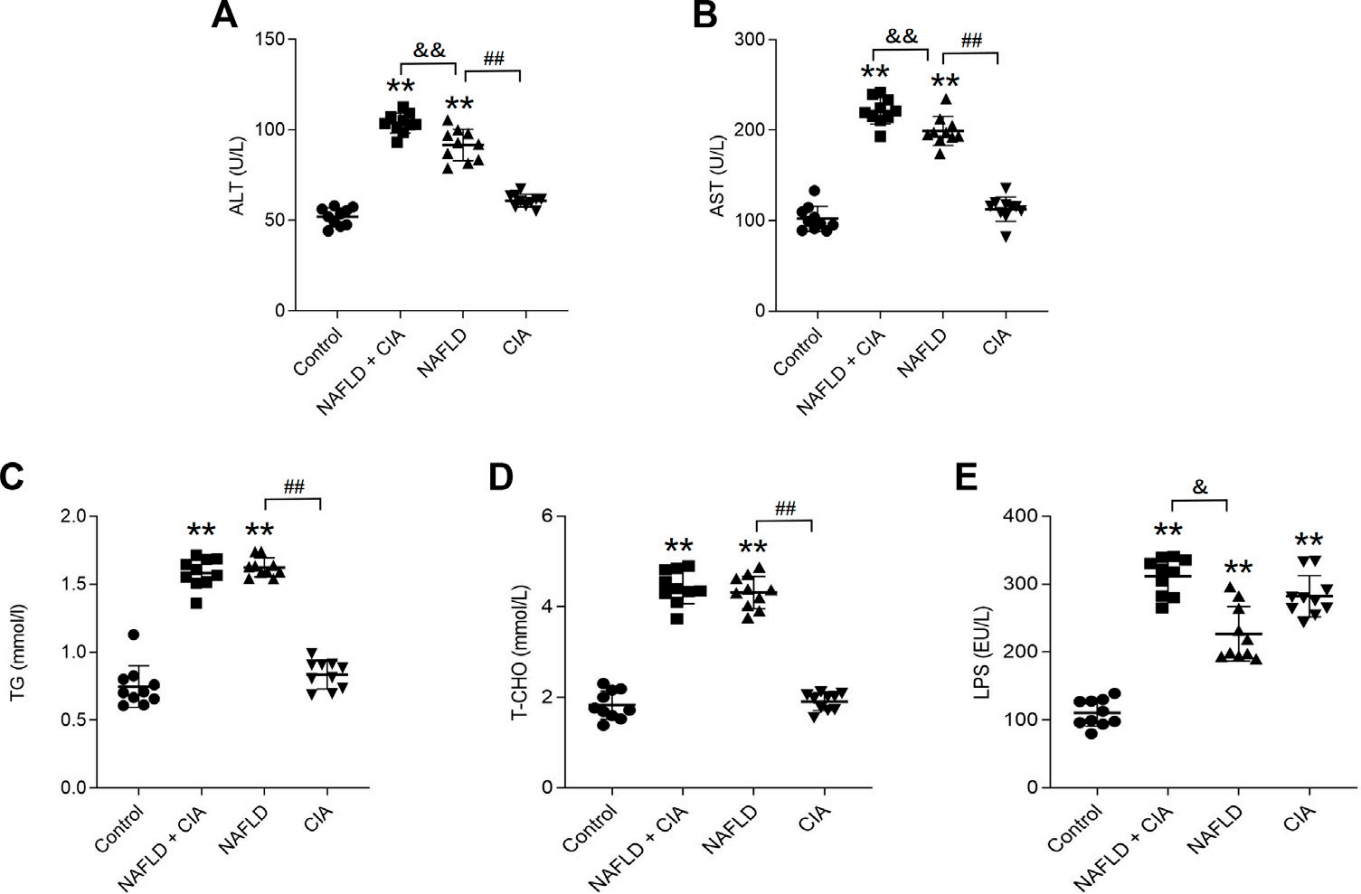
Expression of lipid- and liver function-related biomarkers in the serum of the rats in each group. **(A, B)** ALT and AST (related to liver injury) were detected to evaluate liver injury. **(C, D)** Serum TG and T-CHO levels as determined using respective kits. **(E)** Serum LPS levels were detected by ELISA kit. Data are presented as mean ± SD, *n* = 10. **p < 0.01 *vs*. control. ^##^ *p* < 0.01 *vs*. NAFLD. ^&&^P < 0.01 *vs*. CIA + NAFLD. ^&^P < 0.05 *vs*. CIA + NAFLD.

To evaluate liver function, the serum levels of ALT, AST, TG, T-CHO, and LPS were measured in these rats (Figure 3). As expected, HFD significantly increased all five parameters in both the NAFLD and NAFLD + CIA groups compared with levels in the control group. Interestingly, w the serum TG and T-CHO levels did not differ between the two groups, the ALT and AST levels were greatly increased in the NAFLD + CIA group compared with those in the NAFLD group. LPS levels increased significantly in all groups compared to those in the control group. Furthermore, the serum LPS levels were greatly increased in the NAFLD + CIA group compared with those in the NAFLD group.

### 3.3 Hepatic fibrosis was exacerbated in rats with NAFLD + CIA

**FIGURE 4:**
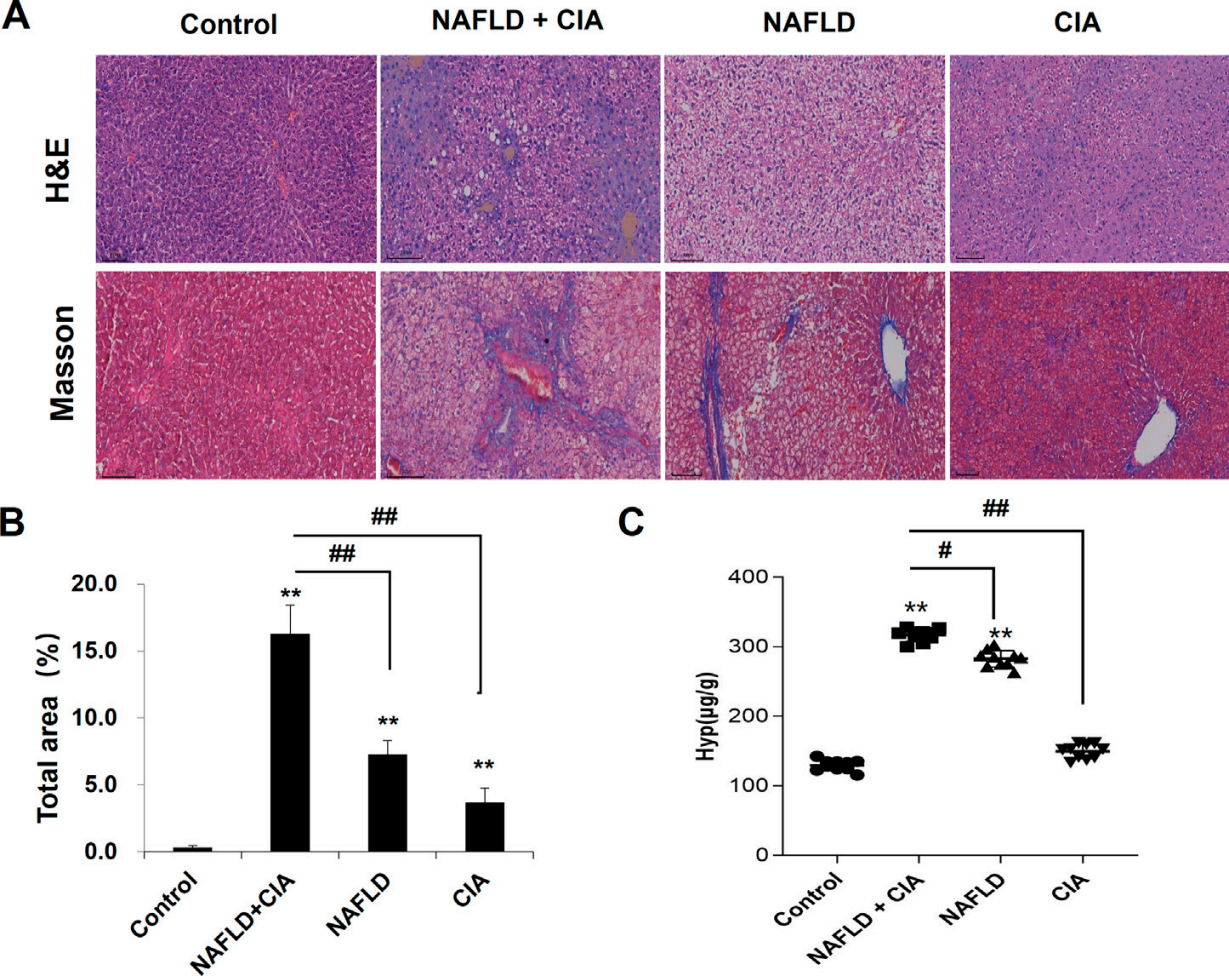
Histopathologic changes of the liver in CIA and NAFLD rats. **(A)** Hepatic histopathology in rats determined by HE and Masson’s trichrome staining. Scale bar = 100 µm. **(B)** Quantitative analysis of Masson’s trichrome staining area. **(C)** The content of Hyp in the liver. Data are presented as mean ± SD, *n* = 10. **p < 0.01 *vs*. control, ^#^ *p* < 0.01 *vs*. CIA + NAFLD, ^##^ *p* < 0.01 *vs*. CIA + NAFLD.

To detect liver morphological changes in the rats, Masson’s trichrome staining was performed in liver sections. As shown in Figure 4, the liver tissue of the rats in the control group had normal hepatic lobule structure with neat hepatic cord arrangement and polygonal-shaped hepatocytes. There was no fibroplasia, inflammatory cell infiltration, and collagen accumulation in these rats. In the CIA group, slight fibroplasia, inflammatory cell infiltration, and collagen accumulation were observed, whereas the structure of the hepatic lobules remained intact in the liver tissues. In the NAFLD and NAFLD + CIA groups, a large number of lipid droplets were found in the cytoplasm, and the nucleus was tilted to one side in the liver tissues of the rats. The hepatic cord arrangement was disrupted, and the hepatocytes showed diffuse steatosis in these animals. Furthermore, the hepatocytes were enlarged, and the cytoplasm was loose. Notably, these morphological changes were greatly exacerbated in the NAFLD + CIA group, compared with that in the NAFLD group. Masson’s trichrome staining indicated that fibrosis was also greatly exacerbated in the former compared with that in the latter group. Hyp is an amino acid unique to collagen, and many diseases can be accompanied by changes in collagen metabolism, causing altered blood, urine, and tissue Hyp levels. Hyp levels in the liver are an important index reflecting the degree of liver fibrosis, and increased Hyp levels in the liver imply increased liver fibrosis (Bedair et al., 2023; Ye et al., 2023). Here, we determined Hyp levels in the liver tissue of the rats and found that the Hyp level was the highest in the NAFLD + CIA group, followed by NAFLD group.

### 3.4 PTRF regulated the hepatic insulin signaling pathway PI3K/Akt in rats with NAFLD + CIA

**FIGURE 5:**
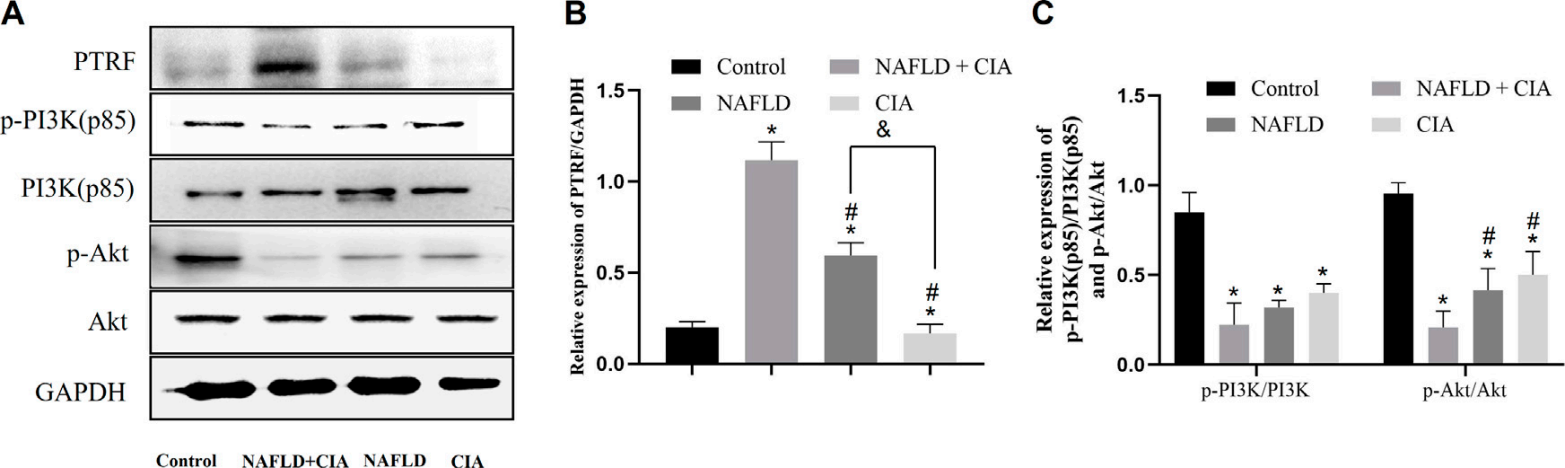
The expression of PTRF and the proteins in the PI3K/Akt signaling pathway in NAFLD/RA rats. **(A)** Western blot analysis of the expression of the proteins PTRF, PI3K (p85), p-PI3K, Akt, and p-Akt in the liver tissues of the rats. **(B)** Densitometric analysis of PTRF expression. GAPDH was used as the internal control. **(C)** Representative quantitative analyses of PI3K (p85), p-PI3K, Akt, and p-Akt levels. Data are reported as mean ± SD (*n* = 3). *p < 0.05 *vs*. control group, ^#^ *p* < 0.05 *vs*. CIA + NAFLD, ^&^P < 0.01 *vs*. NAFLD.

To investigate the pathophysiological role of PTRF in NAFLD, we first measured the expression of the PTRF protein in the hepatic tissue of the rats in each group. Among these rats, PTRF expression was highest in the NAFLD + CIA group (Figure 5A). As aforementioned, the PI3K/Akt pathway is a crucial insulin signaling pathway that affects NAFLD development. Therefore, we assessed the phosphorylation of PI3K and its downstream targets involved in insulin sensitivity in the livers of the rats in the four groups (Figure 5C). HFD-administered rats exhibited significantly impaired insulin signaling, evidenced by decreased PI3K and Akt phosphorylation, compared with that in the rats in the control group, and the lowest expression of p-PI3K and p-AKT proteins in the hepatic tissue of the NAFLD + CIA rats. The expression of total hepatic PI3K and Akt did not vary significantly among the four groups.

### 3.5 AAV-mediated PTRF knockdown enhanced the hepatic insulin signaling pathway PI3K/Akt in rats with NAFLD + CIA

**FIGURE 6:**
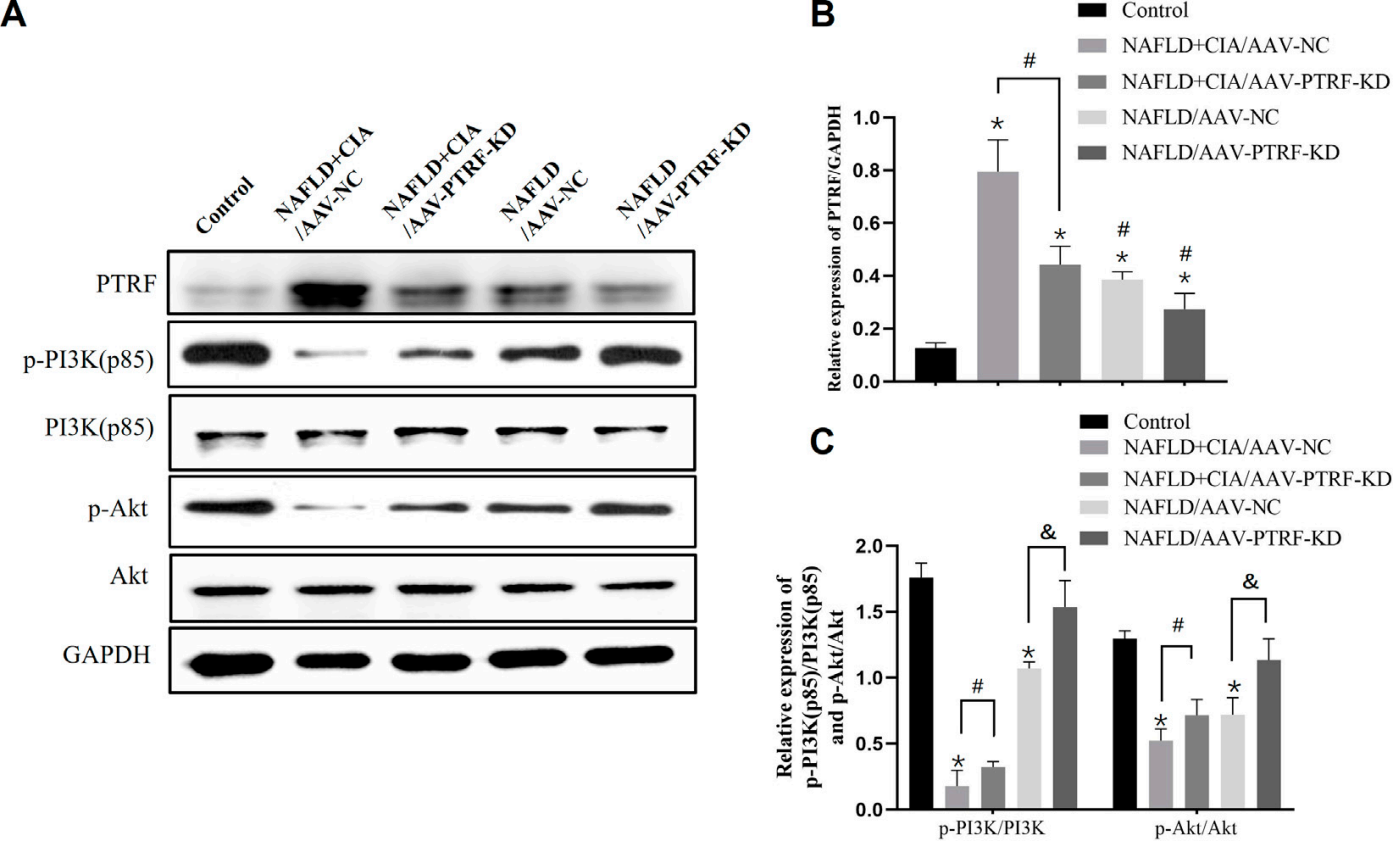
The expression of PTRF and the proteins in the PI3K/Akt signaling pathway in NAFLD/RA rats. **(A)** Western blot analysis of the expression of the proteins PTRF, PI3K (p85), p-PI3K, Akt, and p-Akt in liver tissues. **(B)** Densitometric analysis of PTRF expression. GAPDH was used as the internal control. **(C)** Representative quantitative analyses of PI3K (p85), p-PI3K, Akt, and p-Akt levels. Data are reported as mean ± SD (*n* = 3). *p < 0.05 *vs*. control group, ^#^ *p* < 0.05 NAFLD + CIA/AAV-NC, ^&^ *p* < 0.05 NAFLD/AAV-NC.

Based on the above data, we hypothesized that reduction of PTRF may restore the impaired PI3K/Akt signaling pathway, and thus, reduce TLR4 activation and alleviate hepatic damage in NAFLD + CIA rats. To test this hypothesis, we used the AAV-mediated gene knockdown approach to reduce the expression of PTRF *in vivo*. Among the AAV serotypes, AAV-9 can provide a relatively high efficiency in liver transduction (Nathwani et al., 2009). Thus, we used AAV-9 containing specific shRNA targeting PTFR for gene knockdown. The rats were transfected with AAV-PTRF-KD or control (AAV-NC) vectors through intravenous tail injection. PTRF expression and the activation of its downstream signaling, including the PI3K/Akt pathway, were detected in NAFLD and NAFLD + CIA rats. Western blot results showed that PTRF expression was significantly lower in the NAFLD + CIA/AAV-PTRF-KD and NAFLD/AAV-PTRF-KD groups than in the NAFLD + CIA/AAV-NC and NAFLD/AAV-NC control groups, respectively (Figures 6A, B). As expected, the levels of p-PI3K and p-Akt were significantly decreased in all NAFLD + CIA and NAFLD groups compared with those in the control group. However, AAV-mediated PTRF knockdown increased the phosphorylation of PI3K and Akt compared with that in the AAV-NC group. These results suggested that the suppression of PTRF could maintain the activation of the PI3K/Akt pathway in hepatic tissues.

### 3.6 AAV-mediated PTRF knockdown reduced PTRF/TLR4 interactions in rats with NAFLD + CIA

**FIGURE 7:**
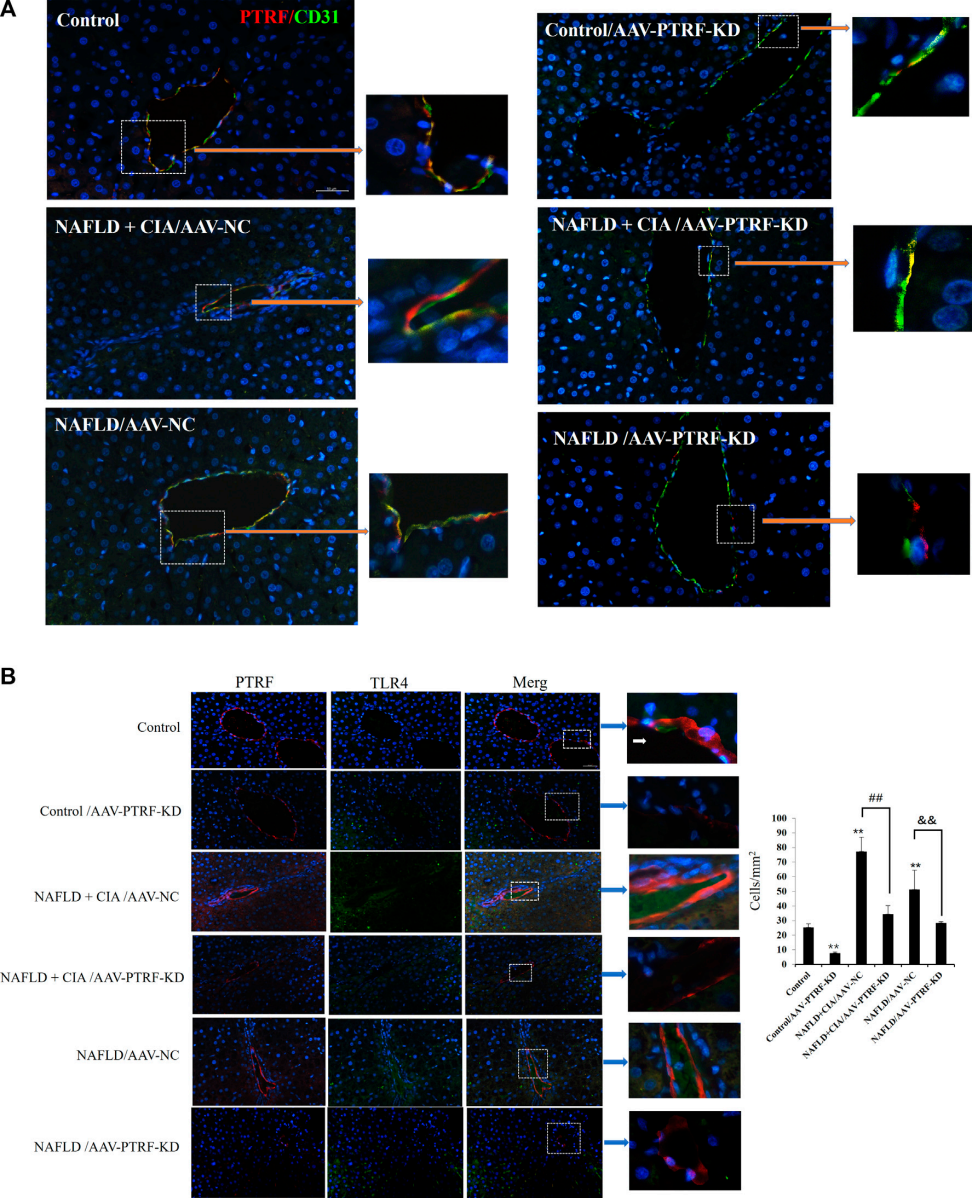
**(A)** Co-localization of PTRF and TLR4 in merged confocal images representing overlays of PTRF (red), CD31 (green), and nuclear staining by DAPI (blue). Scale bar = 50 µm. **(B)** Left: Merged confocal images representing overlays of PTRF (red), TLR4 (green), and nuclear staining by DAPI (blue). Scale bar = 50 µm. Right: Quantitative data of each cell. Data are presented as mean ± SD, *n* = 5. **p < 0.01 *vs*. control, ^##^ *p* < 0.01 *vs*. CIA + NAFLD/AAV-NC, ^&&^ *p* < 0.01 *vs*. NAFLD/AAV-NC.

Confocal imaging was conducted to detect the expression of PTFR in hepatic tissues. As shown in Figure 7A, PTRF was mainly located around small vessels in liver tissues, evidenced by its co-staining with the vascular marker CD31. AAV-PTFR-KD groups showed significantly reduced expression of PTFR. We then examined the cellular localization of PTFR and TLR4 in the rat liver tissues using specific antibodies against PTFR and TLR4, respectively. A strong co-localization pattern for PTRF and TLR4 was observed in the NAFLD + CIA group. Furthermore, AAV-mediated PTRF knockdown resulted in reduced TLR4/PTRF interaction in both NAFLD and NAFLD + CIA rats (Figure 7B).

### 3.7 Reduction of PTRF reduced hepatic pathology in rats with NAFLD + CIA

**FIGURE 8:**
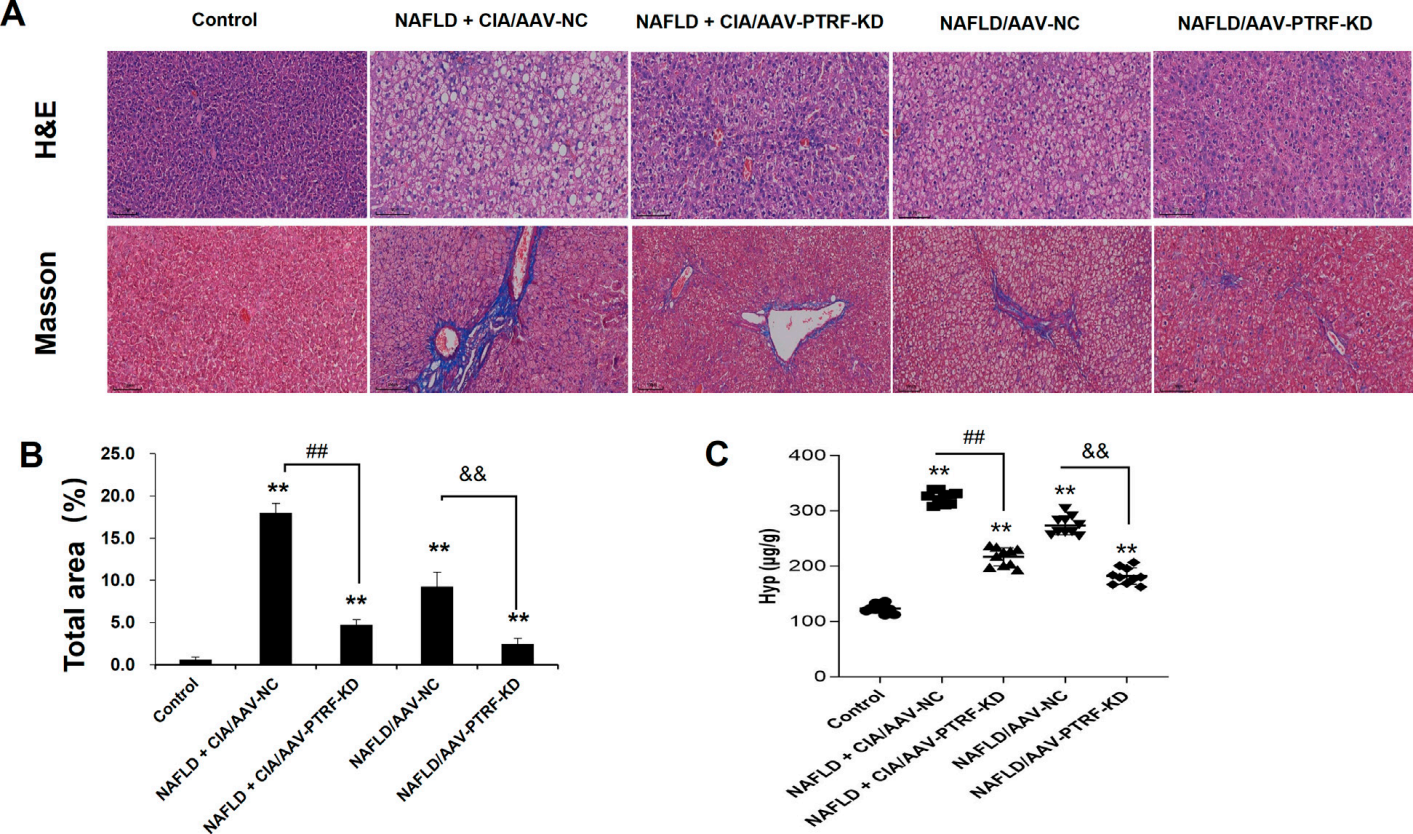
**(A)** Hepatic histopathology in rats determined by HE and Masson’s trichrome staining. Scale bar = 100 µm. **(B)** Quantitative analysis of Masson’s trichrome staining area. **(C)** The content of Hyp in the liver. Data are presented as mean ± SD, *n* = 5. **p < 0.01 *vs*. control, ^##^ *p* < 0.01 *vs*. CIA + NAFLD/AAV-NC, ^&&^ *p* < 0.01 *vs*. NAFLD/AAV-NC.

To evaluate the pathological role of PTRF in the liver tissues of NAFLD + CIA rats, we compared the histological changes in different groups by HE and Masson’s trichrome staining. As shown in Figure 8, intact hepatic lobular structure and morphologically uniform hepatocytes were observed in the liver tissues of control rats. There were no significant deformities, necrosis, or lymphocyte infiltration into the hepatic lobules and portal areas. By contrast, the NAFLD + CIA/AAV-NC and NAFLD/AAV-NC groups exhibited partial hepatic fibrosis and diffuse hepatic steatosis, with minor lymphocyte infiltration in some portal areas and hepatic lobules. Although various degrees of steatosis were still observed in both the NAFLD + CIA/AAV-PTRF KD and NAFLD/AAV-PTRF KD groups, the ballooning degeneration and degree of fibrosis were substantially reduced compared with those in the corresponding AAV-NC groups. The Hyp levels in the livers of rats in each group were consistent with the pathological results. These data suggest that reduction of PTRF can reduce hepatic steatosis and fibrosis in NAFLD rats.

**FIGURE 9:**
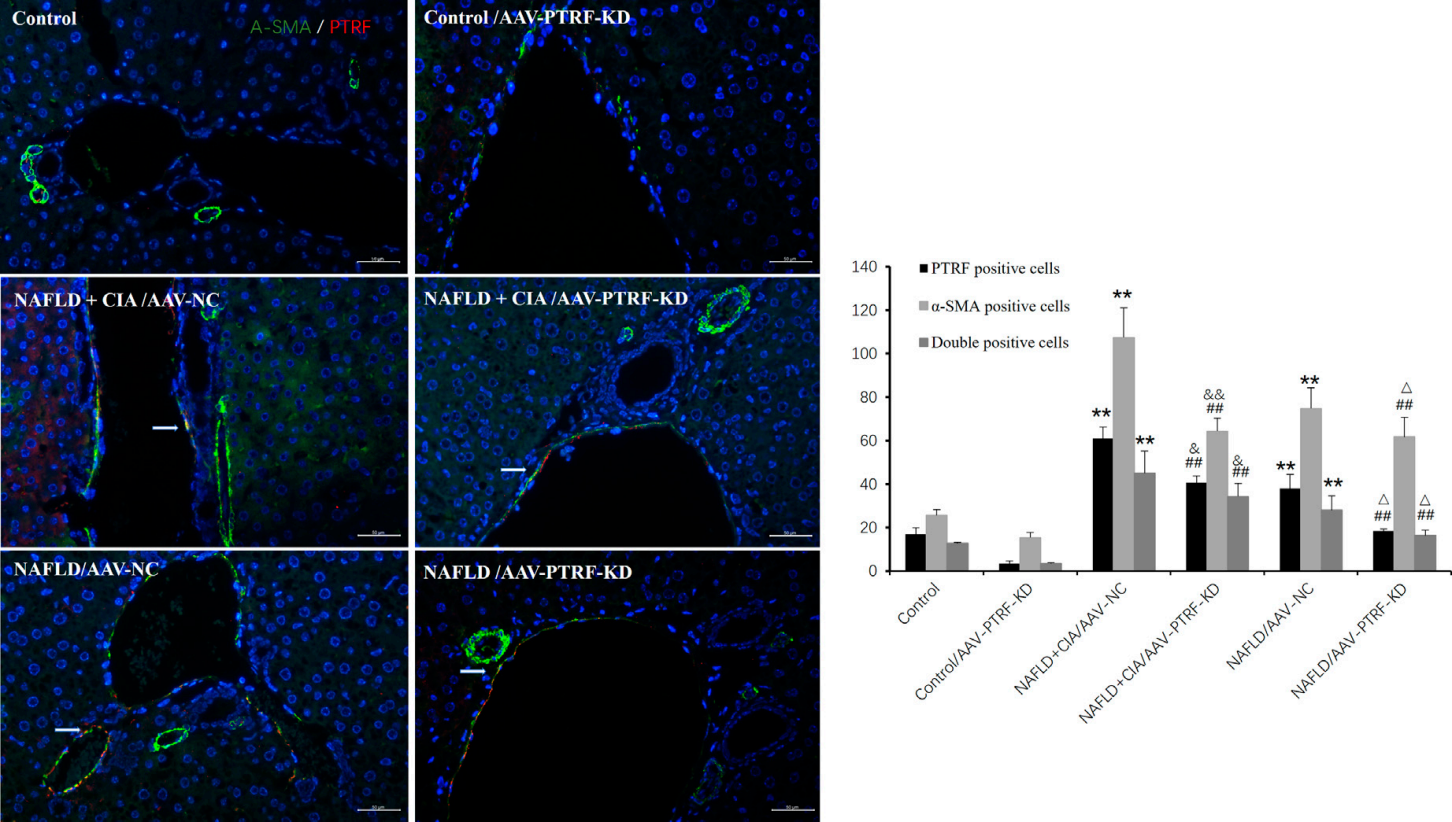
Left: Merged confocal images representing overlays of PTRF (red), α-SMA (green), and nuclear staining by DAPI (blue). Scale bar = 50 µm. Right: Quantitative data of each cell. Data are presented as mean ± SD, *n* = 3. **p < 0.01 *vs*. control, ^##^ *p* < 0.01 *vs*. Control/AAV-PTRF-KD, ^&&^ *p* < 0.01 *vs*. NAFLD + CIA/AAV-NC, ^&^ *p* < 0.05 *vs*. NAFLD + CIA/AAV-NC, ^△^ *p* < 0.05 *vs*. NAFLD/AAV-NC.

Furthermore, immunofluorescence was employed to evaluate the expression of PTRF, α-SMA, and their co-expression during liver fibrosis. Merged confocal images indicated that the AAV-PTRF-KD decreased the expression of PTRF compared to that in the control group (Figure 9). The NAFLD + CIA group exhibited increased α-SMA, PTRF, and their co-expression compared with those in the control group and NAFLD group. However, the NAFLD + CIA/AAV-PTRF-KD group showed a marked reduction of α-SMA, PTRF, and their co-expression in hepatic stellate cells (HSCs) compared with those in the NAFLD + CIA/AAV-NC group. These results also suggest that reduction of PTRF can reduce hepatic steatosis and fibrosis in NAFLD and NAFLD + CIA rats.

## 4 Discussion

Chronic tissue injury leads to fibrosis in many organs, including the liver, lung, kidney, and heart. In chronic liver disease, development of fibrosis is the first step toward organ failure, such as hepatocellular carcinoma (Dasgupta and Manickam, 2023). The extracellular matrix (ECM), which is composed of collagens, elastin, and structural glycoproteins, constitutes the liver “scaffold”. Continuous ECM deposition induces chronic tissue injury of the liver, including fibrogenesis and fibrosis (Tanwar et al., 2020). In the present study, we for the first time showed that the injection of exogenous collagen not only induced serious CIA, but also markedly increased fibrosis in a new compound animal model of CIA and NAFLD. The compound rat model with both NAFLD and CIA was established by administering a HFD and intradermal injection of FCA with collagen. In this compound rat model, HFD exacerbated secondary inflammation in CIA rats (Figures 1, 2) and CIA aggravated liver fibrosis in NAFLD rats (Figures 3, 4).

PTRF is a well-known key protein in the formation of caveola and regulates cellular signaling, endocytosis, and lipid and cholesterol homeostasis. Its pathophysiological role has been best characterized in various cancers (Aung et al., 2011; Wang et al., 2017). However, it is not surprising to see accumulating evidence indicating the involvement of PTRF in liver diseases since this protein regulates phospholipid and triglyceride synthesis and lipid droplet formation in adipocytes. PTRF mutations have been linked to a type of congenital generalized lipodystrophy (Patni and Garg, 2015). PTRF appears to play a critical role in hepatitis, cirrhosis, and hepatocarcinogenesis (Fernandez-Rojo et al., 2013; Gao et al., 2014). It is reported that PTRF stimulates LPS-induced inflammation through the LPS-TLR4- nuclear factor kappa B (NF-κb) pathway, and deletion of PTRF suppresses the inflammatory response and alleviates acute liver injury in mice (Tsai et al., 2018). In PTRF knockout mice, CCl_4_-induced acute liver injury, in terms of necrosis and apoptosis of hepatocytes and ALT level, were dramatically reduced compared with those in wild-type mice (Yang et al., 2021). TLR-4 usually induces the downstream signaling mechanism, such as NF-κB and mitogen activated protein kinases. As mentioned earlier, the hepatic PI3K/Akt axis appears to be the downstream primary insulin signaling pathway affecting NAFLD development (Matsuda et al., 2013). Reduced phosphorylation of PI3K and Akt in hepatocytes contributes to lipid metabolism disorders(Liao et al., 2018). Dysregulation of the PI3K/Akt signaling pathway can increase hepatic lipogenesis and exacerbate free fatty acid flux to the liver, which finally leads to lipolysis (Gu et al., 2019).

Our previous study demonstrated that the TLR4 signaling pathway is stimulated by PTRF (Zhou et al., 2021). In this study, we performed high resolution liver tissue imaging to show that PTRF and TLR4 have a strong co-localization pattern. Moreover, the co-expression of PTRF/TLR4 was decreased when PTRF was knocked down by AAV-PTRF shRNA in NAFLD + CIA rats. Although we cannot provide a conclusion on how PTRF regulates TLR4 based on the present data, we provide indirect evidence that the high expression of both PTRF (in NAFLD) and TLR4 (in CIA) may be the contributing factor for the more severe phenotype of liver dysfunction in the compound NAFLD + CIA rats. Furthermore, we also observed significantly inhibited phosphorylation of PI3K (p85) and Akt in rat livers in NAFLD and compound NAFLD + CIA models. This is likely due to the increased expression of PTRF since knockdown of PTRF could rescue the activation of PI3K in the animal model. Our data also showed that NAFLD + CIA significantly promoted the expression of PTRF and decreased PI3K/Akt activation (Figure 5). Further data showed that AAV9-mediated PTRF knockdown inhibited TLR4 signaling and alleviated hepatic fibrosis in NAFLD + CIA rats (Figures 6–9).

Notably, we observed that PTRF was mainly located around small vessels in liver tissues and co-expressed with CD31, the surface marker of endothelial cells. Further studies showed that PTRF was also co-expressed with α-SMA, which is the main marker of liver fibrosis and mainly expressed in vascular smooth muscle cells and HSCs (Guo et al., 2023; Kim et al., 2023; Wang et al., 2023).

It is well known that matrix components are produced by hepatocytes, endothelial cells, and HSCs, which lie in juxtaposition with the hepatic sinusoid and surround the space of Disse (Schuppan, 1990; Friedman, 2000). Kupffer cells are the resident macrophages of the liver and their major functions include the removal of immune complexes, endotoxins, and excess ECM deposits (Friedman, 2008). HSCs are recognized as the most important cell lineage in the development of liver fibrosis. Furthermore, HSCs interact with other cell types, including hepatocytes, endothelial cells, and Kupfer cells (Giampieri et al., 1981), to promote hepatic fibrosis.

Profibrogenic myofibroblasts can also be derived from portal fibroblasts, epithelial cells, and recruited bone marrow cells that have undergone epithelial to mesenchymal transition (Friedman, 2007). Their embryonic origin is likely to be mesenchymal given that they produce α-SMA in addition to vimentin and desmin when activated (Moreira, 2007).

In line with these reports, this study provided *in vivo* evidence that PTRF played an important role not only in hepatic injury in HFD-induced NAFLD but also in compound NAFLD + CIA rats. Histopathological analysis revealed that AAV-mediated PTRF knockdown could alleviate the increased co-expression of PTRF with CD31/or α-SMA located around small vessels in liver tissues in NAFLD + CIA rats. However, the exact cellular mechanism underlying PTRF regulation of hepatic fibrosis in different cell types, including HSCs, endothelial cells, and Kupfer requires further study.

In summary, this study demonstrated that NAFLD combined with CIA not only causes synovial injury but also enhances non-alcoholic fatty liver fibrosis in rats. PTRF could attenuate the co-expression of PTRF and CD31 in NAFLD + CIA rats by affecting TLR4/PTRF and downstream signaling. These results implied that PTRF could be used as a therapeutic target for diet-induced and collagen-induced hepatic disorders. Future studies on how to target the PTRF/TLR-4/PI3K/Akt axis and clarify its specific roles in different cell types to alleviate liver dysfunction are warranted.

## Data Availability

The original contributions presented in the study are included in the article/Supplementary Material, further inquiries can be directed to the corresponding authors.

## Abbreviations

CIA, Collagen-induced rheumatoid arthritis; FCA, Freund’s complete adjuvant; HFD, High-fat diet; NAFLD, Non-alcoholic fatty liver disease; NASH, Non-alcoholic steatohepatitis; RA, Rheumatoid arthritis; ALT, Alanine aminotransferase; AST, Aspartate aminotransferase; TG, Triglyceride; T-CHO, Total cholesterol; PI3K, Phosphatidylinositol 3-kinase; TLR4, Toll-like receptor 4; LPS, Lipopolysaccharide; PTRF, 1 and transcript release factor; CFA, Complete Freund’s Adjuvant; AAV, Adeno-associated virus; AAV9, Adeno-associated virus serotype 9; HE, Hematoxylin-eosin; DAPI, 4′,6-diamidino-2-phenylindole; Akt, Protein kinase B; SD, Standard deviation.

## References

[B1] Ahmad Khan M. Sarwar A. Rahat R. Ahmed R. S. Umar S. (2020). Stigmasterol protects rats from collagen induced arthritis by inhibiting proinflammatory cytokines. Int. Immunopharmacol. 85, 106642. 10.1016/j.intimp.2020.106642 32470883

[B2] Aung C. S. Hill M. M. Bastiani M. Parton R. G. Parat M. O. (2011). PTRF-cavin-1 expression decreases the migration of PC3 prostate cancer cells: Role of matrix metalloprotease 9. Eur. J. Cell Biol. 90 (2-3), 136–142. 10.1016/j.ejcb.2010.06.004 20732728

[B4] Bai L. Lyu Y. Shi G. Li K. Huang Y. Ma Y. (2020). Polymerase I and transcript release factor transgenic mice show impaired function of hematopoietic stem cells. Aging (Albany NY) 12 (20), 20152–20162. 10.18632/aging.103729 33087586 PMC7655181

[B5] Bedair A. F. Wahid A. El-Mezayen N. S. Afify E. A. (2023). Nicorandil reduces morphine withdrawal symptoms, potentiates morphine antinociception, and ameliorates liver fibrosis in rats. Life Sci. 319, 121522. 10.1016/j.lfs.2023.121522 36822314

[B6] Bellentani S. Scaglioni F. Marino M. Bedogni G. (2010). Epidemiology of non-alcoholic fatty liver disease. Dig. Dis. 28 (1), 155–161. 10.1159/000282080 20460905

[B7] Calvo M. Tebar F. Lopez-Iglesias C. Enrich C. (2001). Morphologic and functional characterization of caveolae in rat liver hepatocytes. Hepatology 33 (5), 1259–1269. 10.1053/jhep.2001.23937 11343255

[B8] Csak T. Ganz M. Pespisa J. Kodys K. Dolganiuc A. Szabo G. (2011). Fatty acid and endotoxin activate inflammasomes in mouse hepatocytes that release danger signals to stimulate immune cells. Hepatology 54 (1), 133–144. 10.1002/hep.24341 21488066 PMC4158408

[B3] Dasgupta T. Manickam V. (2023). Fibrosis in Liver and Pancreas: a Review on Pathogenic Significance, Diagnostic Options, and Current Management Strategies. Inflammation 46 (3), 824–834. 10.1007/s10753-022-01776-0 36595108

[B9] Ding S. Y. Lee M. J. Summer R. Liu L. Fried S. K. Pilch P. F. (2014). Pleiotropic effects of cavin-1 deficiency on lipid metabolism. J. Biol. Chem. 289 (12), 8473–8483. 10.1074/jbc.M113.546242 24509860 PMC3961672

[B10] Fang Y. Shen Z. Y. Zhan Y. Z. Feng X. C. Chen K. L. Li Y. S. (2019). CD36 inhibits beta-catenin/c-myc-mediated glycolysis through ubiquitination of GPC4 to repress colorectal tumorigenesis. Nat. Commun. 10 (1), 3981. 10.1038/s41467-019-11662-3 31484922 PMC6726635

[B11] Farrell G. C. Larter C. Z. (2006). Nonalcoholic fatty liver disease: From steatosis to cirrhosis. Hepatology 43 (2), S99–S112. 10.1002/hep.20973 16447287

[B12] Fernandez-Rojo M. A. Gongora M. Fitzsimmons R. L. Martel N. Martin S. D. Nixon S. J. (2013). Caveolin-1 is necessary for hepatic oxidative lipid metabolism: Evidence for crosstalk between caveolin-1 and bile acid signaling. Cell Rep. 4 (2), 238–247. 10.1016/j.celrep.2013.06.017 23850288

[B13] Fernandez-Rojo M. A. Ramm G. A. (2016). Caveolin-1 function in liver physiology and disease. Trends Mol. Med. 22 (10), 889–904. 10.1016/j.molmed.2016.08.007 27633517

[B14] Friedman S. L. (2007). Liver fibrosis: From mechanisms to treatment. Gastroenterol. Clin. Biol. 31 (10), 812–814. 10.1016/s0399-8320(07)73970-2 18166858

[B15] Friedman S. L. (2008). Mechanisms of hepatic fibrogenesis. Gastroenterology 134 (6), 1655–1669. 10.1053/j.gastro.2008.03.003 18471545 PMC2888539

[B16] Friedman S. L. (2000). Molecular regulation of hepatic fibrosis, an integrated cellular response to tissue injury. J. Biol. Chem. 275 (4), 2247–2250. 10.1074/jbc.275.4.2247 10644669

[B17] Gao L. Zhou Y. Zhong W. Zhao X. Chen C. Chen X. (2014). Caveolin-1 is essential for protecting against binge drinking-induced liver damage through inhibiting reactive nitrogen species. Hepatology 60 (2), 687–699. 10.1002/hep.27162 24710718

[B18] Giampieri M. P. Jezequel A. M. Orlandi F. (1981). The lipocytes in normal human liver. A quantitative study. Digestion 22 (4), 165–169. 10.1159/000198640 7308589

[B19] Gu Y. Gao L. Han Q. Li A. Yu H. Liu D. (2019). GSK-3β at the crossroads in regulating protein synthesis and lipid deposition in zebrafish. Cells 8 (3), 205. 10.3390/cells8030205 30823450 PMC6468354

[B20] Guo J. Cao X. Ma X. Hao C. Wu L. Zhang M. (2020). Tumor necrosis factor receptor-associated factor 6 (TRAF6) inhibition modulates bone loss and matrix metalloproteinase expression levels in collagen-induced rheumatoid arthritis rat. Ann. Palliat. Med. 9 (6), 4017–4028. 10.21037/apm-20-1894 33183053

[B21] Guo M. Wang Z. Dai J. Fan H. Yuan N. Gao L. (2023). Glycyrrhizic acid alleviates liver fibrosis *in vitro* and *in vivo* via activating CUGBP1-mediated IFN-γ/STAT1/Smad7 pathway. Phytomedicine 112, 154587. 10.1016/j.phymed.2022.154587 36805480

[B22] Jia L. Vianna C. R. Fukuda M. Berglund E. D. Liu C. Tao C. (2014). Hepatocyte Toll-like receptor 4 regulates obesity-induced inflammation and insulin resistance. Nat. Commun. 5, 3878. 10.1038/ncomms4878 24815961 PMC4080408

[B23] Kim K. E. Lee J. Shin H. J. Jeong E. A. Jang H. M. Ahn Y. J. (2023). Lipocalin-2 activates hepatic stellate cells and promotes nonalcoholic steatohepatitis in high-fat diet-fed Ob/Ob mice. Hepatology 77 (3), 888–901. 10.1002/hep.32569 35560370 PMC9936980

[B24] Liao X. Song L. Zhang L. Wang H. Tong Q. Xu J. (2018). LAMP3 regulates hepatic lipid metabolism through activating PI3K/Akt pathway. Mol. Cell Endocrinol. 470, 160–167. 10.1016/j.mce.2017.10.010 29056532

[B25] Matsuda S. Kobayashi M. Kitagishi Y. (2013). Roles for PI3K/AKT/PTEN pathway in cell signaling of nonalcoholic fatty liver disease. ISRN Endocrinol. 2013, 472432. 10.1155/2013/472432 23431468 PMC3570922

[B26] Maurice J. Manousou P. (2018). Non-alcoholic fatty liver disease. Clin. Med. (Lond) 18 (3), 245–250. 10.7861/clinmedicine.18-3-245 29858436 PMC6334080

[B27] Moreira R. K. (2007). Hepatic stellate cells and liver fibrosis. Arch. Pathol. Lab. Med. 131 (11), 1728–1734. 10.5858/2007-131-1728-hscalf 17979495

[B28] Mori S. Arima N. Ito M. Fujiyama S. Kamo Y. Ueki Y. (2018). Non-alcoholic steatohepatitis-like pattern in liver biopsy of rheumatoid arthritis patients with persistent transaminitis during low-dose methotrexate treatment. PLoS One 13 (8), e0203084. 10.1371/journal.pone.0203084 30142184 PMC6108522

[B29] Nathwani A. C. Cochrane M. McIntosh J. Ng C. Y. Zhou J. Gray J. T. (2009). Enhancing transduction of the liver by adeno-associated viral vectors. Gene Ther. 16 (1), 60–69. 10.1038/gt.2008.137 18701909 PMC2615795

[B30] Patni N. Garg A. (2015). Congenital generalized lipodystrophies--new insights into metabolic dysfunction. Nat. Rev. Endocrinol. 11 (9), 522–534. 10.1038/nrendo.2015.123 26239609 PMC7605893

[B31] Ruderman E. M. Crawford J. M. Maier A. Liu J. J. Gravallese E. M. Weinblatt M. E. (1997). Histologic liver abnormalities in an autopsy series of patients with rheumatoid arthritis. Br. J. Rheumatol. 36 (2), 210–213. 10.1093/rheumatology/36.2.210 9133932

[B32] Sanyal A. J. Friedman S. L. McCullough A. J. Dimick-Santos L. United States F. (2015). Challenges and opportunities in drug and biomarker development for nonalcoholic steatohepatitis: Findings and recommendations from an American association for the study of liver diseases-U.S. Food and drug administration joint workshop. Hepatology 61 (4), 1392–1405. 10.1002/hep.27678 25557690 PMC4900161

[B33] Schuppan D. (1990). Structure of the extracellular matrix in normal and fibrotic liver: Collagens and glycoproteins. Semin. Liver Dis. 10 (1), 1–10. 10.1055/s-2008-1040452 2186485

[B34] Selmi C. De Santis M. Gershwin M. E. (2011). Liver involvement in subjects with rheumatic disease. Arthritis Res. Ther. 13 (3), 226. 10.1186/ar3319 21722332 PMC3218873

[B35] Shen C. Ma W. Ding L. Li S. Dou X. Song Z. (2018). The TLR4-IRE1α pathway activation contributes to palmitate-elicited lipotoxicity in hepatocytes. J. Cell Mol. Med. 22 (7), 3572–3581. 10.1111/jcmm.13636 29673059 PMC6010797

[B36] Tanwar S. Rhodes F. Srivastava A. Trembling P. M. Rosenberg W. M. (2020). Inflammation and fibrosis in chronic liver diseases including non-alcoholic fatty liver disease and hepatitis C. World J. Gastroenterol. 26 (2), 109–133. 10.3748/wjg.v26.i2.109 31969775 PMC6962431

[B37] Tsai T. H. Tam K. Chen S. F. Liou J. Y. Tsai Y. C. Lee Y. M. (2018). Deletion of caveolin-1 attenuates LPS/GalN-induced acute liver injury in mice. J. Cell Mol. Med. 22 (11), 5573–5582. 10.1111/jcmm.13831 30134043 PMC6201225

[B38] Wang F. Zheng Y. Orange M. Yang C. Yang B. Liu J. (2017). PTRF suppresses the progression of colorectal cancers. Oncotarget 8 (30), 48650–48659. 10.18632/oncotarget.9424 27203393 PMC5564714

[B39] Wang J. Yang Y. Sun F. Luo Y. Yang Y. Li J. (2023). ALKBH5 attenuates mitochondrial fission and ameliorates liver fibrosis by reducing Drp1 methylation. Pharmacol. Res. 187, 106608. 10.1016/j.phrs.2022.106608 36566000

[B40] Wang X. Yan X. Wang F. Ge F. Li Z. (2018). Role of methotrexate chronotherapy in collagen-induced rheumatoid arthritis in rats. Z Rheumatol. 77 (3), 249–255. 10.1007/s00393-016-0236-6 27900440 PMC5884908

[B41] Yang Z. Zhang J. Wang Y. Lu J. Sun Q. (2021). Caveolin-1 deficiency protects mice against carbon tetrachloride-induced acute liver injury through regulating polarization of hepatic macrophages. Front. Immunol. 12, 713808. 10.3389/fimmu.2021.713808 34434195 PMC8380772

[B42] Ye J. Chen J. Li Y. Sun L. Lu H. (2023). Hepatocyte-specific knockout of HIF-2α cannot alleviate carbon tetrachloride-induced liver fibrosis in mice. PeerJ 11, e15191. 10.7717/peerj.15191 37033734 PMC10078453

[B43] Zhai Y. Shen X. D. O'Connell R. Gao F. Lassman C. Busuttil R. W. (2004). Cutting edge: TLR4 activation mediates liver ischemia/reperfusion inflammatory response via IFN regulatory factor 3-dependent MyD88-independent pathway. J. Immunol. 173 (12), 7115–7119. 10.4049/jimmunol.173.12.7115 15585830

[B44] Zheng Y. Lee S. Liang X. Wei S. Moon H. G. Jin Y. (2013). Suppression of PTRF alleviates the polymicrobial sepsis induced by cecal ligation and puncture in mice. J. Infect. Dis. 208 (11), 1803–1812. 10.1093/infdis/jit364 23908488 PMC3814834

[B45] Zhou H. H. Zhang Y. M. Zhang S. P. Xu Q. X. Tian Y. Q. Li P. (2021). Suppression of PTRF alleviates post-infectious irritable bowel syndrome via downregulation of the TLR4 pathway in rats. Front. Pharmacol. 12, 724410. 10.3389/fphar.2021.724410 34690766 PMC8529073

[B46] Zhu L. Li D. Yang X. (2023). Gut metabolomics and 16S rRNA sequencing analysis of the effects of arecoline on non-alcoholic fatty liver disease in rats. Front. Pharmacol. 14, 1132026. 10.3389/fphar.2023.1132026 37050898 PMC10083296

